# Breaking enhancers to gain insights into developmental defects

**DOI:** 10.7554/eLife.88187

**Published:** 2023-07-27

**Authors:** Daniel A Armendariz, Anjana Sundarrajan, Gary C Hon

**Affiliations:** 1 Cecil H. and Ida Green Center for Reproductive Biology Sciences, University of Texas Southwestern Medical Center Dallas United States; 2 Hamon Center for Regenerative Science and Medicine, University of Texas Southwestern Medical Center Dallas United States; 3 Lyda Hill Department of Bioinformatics, Department of Obstetrics and Gynecology, University of Texas Southwestern Medical Center Dallas United States; Seoul National University Republic of Korea; Seoul National University Republic of Korea

**Keywords:** genomics, genetic diseases, CRISPR, development

## Abstract

Despite ground-breaking genetic studies that have identified thousands of risk variants for developmental diseases, how these variants lead to molecular and cellular phenotypes remains a gap in knowledge. Many of these variants are non-coding and occur at enhancers, which orchestrate key regulatory programs during development. The prevailing paradigm is that non-coding variants alter the activity of enhancers, impacting gene expression programs, and ultimately contributing to disease risk. A key obstacle to progress is the systematic functional characterization of non-coding variants at scale, especially since enhancer activity is highly specific to cell type and developmental stage. Here, we review the foundational studies of enhancers in developmental disease and current genomic approaches to functionally characterize developmental enhancers and their variants at scale. In the coming decade, we anticipate systematic enhancer perturbation studies to link non-coding variants to molecular mechanisms, changes in cell state, and disease phenotypes.

## Introduction

Enhancers are regulatory elements that drive development and lineage specification through temporal and cell-type-specific control of gene expression (Banerji et al., 1981). Thus, it is not surprising that enhancer dysregulation has been frequently implicated in developmental diseases (termed ‘enhanceropathies’). Whole genome sequencing (**WGS**) and genome-wide association studies (**GWAS**) have highlighted an abundance of disease-associated genetic variants at enhancers (Gusev et al., 2014; Parker et al., 2013). It is thought that these variants alter the activity of enhancers, impact gene expression programs, and ultimately contribute to disease risk and pathogenesis (Kleinjan and van Heyningen, 2005).

Several notable developmental enhanceropathies highlight key concepts. First, unlike protein-coding variants, the impact of genetic variants on enhancers may not be obvious. For example, point mutations of an enhancer of the sonic hedgehog (SHH) gene cause the developmental malformation, polydactyly (Lettice et al., 2017; Lettice et al., 2003). This enhancer was initially mapped to an intron of the LMBR1 gene through breakpoint analysis in a patient with preaxial polydactyly (Lettice et al., 2003). Functional analysis of the enhancer sequence in transgenic mouse embryos showed that the regulatory element’s activity overlapped with SHH expression in the zone of polarizing activity (**ZPA**) during early limb specification. Remarkably, this enhancer is over 1 Mb away from the SHH gene promoter and does not regulate the expression of several genes in closer proximity (Williamson et al., 2016). This example illustrates the potential for non-coding enhancer variants to cause developmental defects owing to their roles in early cell specification, by regulating distally located genes. This example also highlights the importance of 3D genome architecture in gene regulation and the interpretation of enhanceropathy mechanisms. Other examples include a long-range enhancer of IRX3 with obesity-associated variants in the brain (Smemo et al., 2014) and a cardiometabolic risk-associated enhancer that regulates the *FST* gene ∼522 kb away in the liver (Civelek et al., 2017). This topic of long-range gene regulation is more extensively covered in recent reviews (Razin et al., 2023; Vermunt et al., 2019).

Second, expanding on the first concept, enhancers can contribute to disease through indirect and cell-type specific mechanisms. For example, a risk-associated enhancer for the persistence of fetal hemoglobin alters the expression of the transcriptional repressor BCL11A (Bauer et al., 2013). However, it is not BCL11A per se, but its target HbF (fetal hemoglobin) that is directly responsible for the phenotype (Basak et al., 2015). Importantly, this gene regulatory event only manifests in hematopoietic cells. Third, enhanceropathies can phenocopy genetic variants. Congenital heart defects (CHD) are a widespread class of cardiac defects with multiple gene drivers (Fahed et al., 2013). One of the more well-known drivers is TBX5, with exon mutations associated with a variety of CHD-related complications such as Holt-Oram Syndrome and atrial/septal defects (Bruneau et al., 2001; McDermott et al., 2005). Sequencing studies in CHD patients identified several genetic variants, a handful of which ablated the activity of a TBX5 enhancer (Smemo et al., 2012). This observation suggests that TBX5 enhanceropathies can contribute to CHD by reducing TBX5 expression, and phenocopies haploinsufficiency of TBX5 caused by gene mutations.

**Figure 1.**
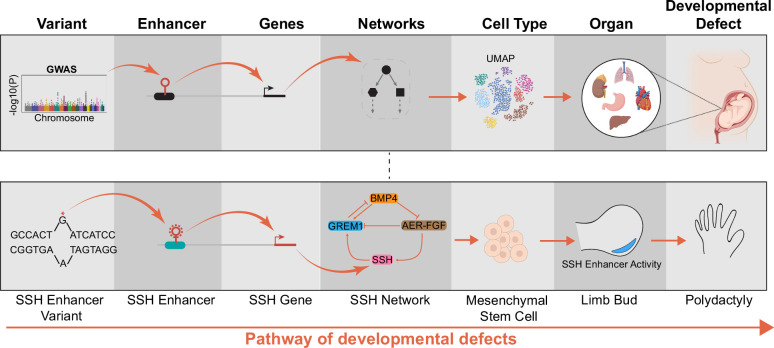
Pathway of how enhancer variants cause developmental defects. The functional characterization of enhancer variants is a multi-step process linking genotypes to molecular phenotypes (target genes and networks), cellular phenotypes (cell state, morphology), and organismal phenotypes (developmental defect) (top row). A genetic variant of a Sonic Hedgehog enhancer driving polydactyly is one well-characterized example (bottom row). However, the role of most cis-regulatory elements in developmental disease remains unclear.

The culmination of genetic studies has identified enhancer variants as major factors in the human diseases and underscores the role of disrupted spatiotemporal gene regulation in developmental disease. However, as the above examples indicate, the assignment of risk variants to the mechanisms of developmental diseases is often non-trivial. A key challenge in the field is to directly perturb enhancers, their variants, and associated genes in the appropriate cell type to identify their impacts on molecular, cellular, and organismal phenotypes that are relevant to developmental diseases (Figure 1). Here, we will review the development of recent genomic technologies to accomplish this ambitious goal and anticipate future challenges along three key dimensions: genomic perturbations, cellular systems, and the phenotypic readouts.

### Functional evaluation of enhancer elements

Since disease-associated variants overlap enhancers, one approach to gain clues to the mechanisms of variants is to first perform a functional analysis of whole enhancer elements. This approach has several advantages. First, diverse tools have been developed to measure enhancer function at scale, including massively parallel reporter assays and CRISPR-based screening approaches (Table 1). Recent approaches have coupled these technologies with single-cell assays to further increase the scale and resolution of enhancer characterization (Dixit et al., 2016). Second, by focusing analyses on the small subset of enhancers that overlap variants (out of the universe of millions), this approach significantly reduces the set of enhancers to functionally test. While a given developmental state may have thousands of active enhancers, many will not be relevant to the disease. However, it is important to note that measuring the activity of an entire enhancer does not directly answer how a disease variant functions. Nonetheless, this approach can serve to prioritize enhancers for downstream variant-level analyses, which are more challenging to perform and lower throughput.

**Table 1.** Summary of genomic approaches to characterize enhancers and variants.

Approach	Application	Pros	Cons	Example studies
VISTA	Measures in vivo enhancer reporter activity in transgenic mice.	in vivo and spatial readouts of enhancer activity	single time point (E11.5), low throughput	Pennacchio et al., 2006; Visel et al., 2007
Massively parallel reporter assays (MPRA), Self-transcribing active regulatory region (STARR)-Seq	Measures the activity of enhancer sequences and variants with high throughput reporter assays. Single-cell MPRA gives readouts on cell-specific enhancer activity.	very high throughput, variant-level activity	lacks endogenous genomic context, readouts can depend on the design of reporter constructs	Inoue et al., 2019; Kircher et al., 2019; Arnold et al., 2013; Zhao et al., 2023; Lalanne et al., 2022
CRISPR screen	Measures endogenous activity of enhancer by perturbating sequences with CRISPR/Cas9, using sgRNA dropout as a phenotypic readout.	high throughput, endogenous genomic context	requires a selectable phenotype, the readout is only sgRNA abundance	Sanjana et al., 2016; Korkmaz et al., 2016
CRISPRi FlowFISH, HCR FlowFISH	Measures endogenous enhancer activity on the expression of candidate genes with high sensitivity.	medium throughput, endogenous genomic context, sensitive transcriptional readout	only a small number of genes can be measured in each experiment	Fulco et al., 2016; Reilly et al., 2021
Single-cell CRISPRi screen	Measures endogenous enhancer activity on transcriptome-wide phenotypes.	high throughput, endogenous genomic context, transcriptome-wide readout	low sensitivity for lowly expressed genes or enhancers with modest effects; expensive	Genga et al., 2019; Armendariz et al., 2022
Base editing screen	Measures endogenous activity of enhancer variants after high throughput base editing.	variant-level perturbations more relevant to disease modeling, endogenous genomic context	some base substitutions incompatible with current base editors, limited editing window restricts sgRNA design; modest effect sizes	Martin-Rufino et al., 2023; Chen et al., 2022
merFISH, seqFISH, osmFISH	Measures spatial RNA expression with high sensitivity.	spatial context, sensitive readout of many transcripts	existing screens are low throughput, expensive, specialized equipment	Xie et al., 2017; Eng et al., 2019; Codeluppi et al., 2018
Imaging screen (Cell Painting, optical)	Measures morphological phenotypes after perturbation.	spatial readout, morphological phenotypes of multiple cellular components	enhancer perturbations may not cause morphological phenotypes; lacks gene expression readout; limited cell type compatibility	Bray et al., 2016

### Reporter assays for analysis of enhancer elements during in vivo development

Since enhancer activity is cell-type and developmental time specific, an important initial step is to define active enhancers in a developmental system. Chromatin-based approaches have been widely adopted for this purpose. In particular, recent applications of single-cell ATAC-Seq in developmental systems have enabled the identification of thousands of potential enhancers at a cell-type specific level (Cusanovich et al., 2018a; Cusanovich et al., 2018b; Domcke et al., 2020; Gao et al., 2022). Chromosome conformation capture assays have also been extensively applied to identify enhancers that engage in 3D chromatin interactions with putative target genes (Lu et al., 2020; Ron et al., 2017). For focused discussion on these topics, we refer the reader to other reviews (Gasperini et al., 2020; Razin et al., 2023; Shlyueva et al., 2014; Vermunt et al., 2019).

While epigenetic signatures can be used to identify putative enhancer elements, functional assays are needed to definitively demonstrate an enhancer’s spatiotemporal activity during in vivo development. Reporter assays are simple and flexible tools that have addressed this gap in enhancer functional characterization. In a reporter assay, enhancer activity is assessed by cloning a putative enhancer downstream of a minimal promoter driving expression of a reporter gene (Stanojevic et al., 1991). For example, Conrad and Botchan used a reporter assay to discover the first enhancer as an element of the SV40 genome that drove the expression of a B-globin reporter gene in HeLa cells (Conrad and Botchan, 1982). However, reporters test enhancers outside of their native genomic context. A key development has been the extension of reporter assays to characterize the activity of enhancers in vivo (Hammer et al., 1987; Johnson et al., 1989; Pinkert et al., 1987; Swift et al., 1984). Imaging-based approaches, such as those incorporating luciferase or lacZ, have also enabled high-resolution spatiotemporal characterization of enhancer activity during mammalian development (Kvon et al., 2020; Pennacchio et al., 2006; Smemo et al., 2012). Notably, the VISTA Enhancer browser now documents thousands of enhancers that have been experimentally tested using reporter assays in embryonic mice (Visel et al., 2007). These detailed images of stained embryos provided by in vivo reporter assays demonstrate the exquisite spatiotemporal specificity of enhancers in development. However, the scale of traditional reporter assays remains limited to a handful of targets.

### High throughput functional characterization of developmental enhancers

Massively parallel reporter assays (**MPRA**) use next-generation sequencing to measure enhancer activity at scale (Melnikov et al., 2012; Patwardhan et al., 2009). In an MPRA, thousands of DNA sequences are cloned into a reporter construct upstream of a minimal promoter, a fluorescent marker, and a barcode. By sequencing the barcodes and measuring abundance, the level of expression provided by each enhancer can be determined (Inoue and Ahituv, 2015). Innoue et al., applied an MPRA to characterize over 2,000 enhancers throughout the differentiation of human embryonic stem cells toward neural progenitors (Inoue et al., 2019). By collecting samples at multiple time points, the authors demonstrated the temporal specificity of enhancers. Notably, enhancer activity in the exogenous reporter correlated with epigenetic hallmarks found at the endogenous enhancers including ATAC-seq and H3K27ac, and target gene expression. As MPRAs test thousands of enhancers, they also reveal patterns of transcription factor binding and the features that drive enhancer activity (Smith et al., 2013). For example, Inoue et al., found that regulatory activity depended primarily on chromatin context (Inoue et al., 2019). The sequence of the regulatory elements, including binding motifs, primarily dictated whether these regions acted to increase or decrease transcription. STARR-seq is another massively parallel reporter assay that systematically tests all genomic fragments for enhancer activity (Arnold et al., 2013). An adaptation of STARR-seq has been applied in vivo to assess 408 sequences for enhancer activity in the early mouse brain (Lambert et al., 2021). This approach has the advantage of measuring the spatial activity of enhancers in vivo.

Traditional MPRAs are limited to characterizing putative enhancers in a uniform cell population. To address this issue, MPRAs have recently been combined with single-cell RNA-Seq (**scRNA-seq**) to characterize enhancer activity in heterogeneous cell systems (Lalanne et al., 2022; Zhao et al., 2023). Lalanne et al characterized 213 putative regulatory elements in mouse embryonic stem cell-derived embryoid bodies generating the three germ layers (Lalanne et al., 2022). The authors identified an enhancer of Lamc1 which exhibits pleiotropic activity in two populations, in contrast to the Lamc1 gene which is expressed in a single cell type. This discordant regulation suggests that the expression of Lamc1 is not strictly dependent on the activity of its nearby enhancer. These studies highlight the power of single-cell technologies to address enhancer cell-type specificity and their ability to reveal regulatory networks. Thus, MPRAs expand the throughput of reporter assays and can be used to identify features of enhancers active in particular developmental systems.

Despite the widespread use, reporter assays have several limitations. First, the choice of the expression vector can produce dramatic differences in enhancer-mediated expression that can complicate interpretation (Lungu-Mitea and Lundqvist, 2020). For example, Lungu-Mitea et al., queried multiple reporter backbones and identified an induction fold difference of 10 between two backbones. Second, the length of the insert selected, often limited by DNA synthesis technologies, may not capture all the functional sequences of an enhancer (Romanov et al., 2021). Klein et al., performed an MPRA containing different enhancer lengths and found that results tended to correlate poorly between lengths (Klein et al., 2020). Finally, since reporter assays are exogenous, they do not recapitulate endogenous enhancer function. For example, in a screen of over 2000 DNA sequences, Inoue et al., observed that half of the identified putative enhancers did not correlate with endogenous chromatin signatures (Inoue et al., 2019). Thus, while reporter systems have the flexibility and scalability to assess thousands of sequences for enhancer activity, other methods are required to determine the developmental relevance of enhancers in their native genomic context.

### Genome engineering for endogenous analysis of enhancers in development

Endogenous perturbations of enhancers in their genomic context are required to understand the role of these regulatory elements in development. TALENs, which are programmable nucleases that can be recruited to user-specified genomic loci, were initially applied to this question (Boch et al., 2009; Moscou and Bogdanove, 2009). Wang et al. directed TALENs to an enhancer in which lupus-associated variants had been identified (Wang et al., 2016). The introduction of variants at this enhancer disrupted promoter interaction with TNFAIP3 and ultimately led to the downregulation of the downstream autoimmune gene NF-κB. While TALENs allow for targeted genetic perturbations, they are too difficult to scale for genetic screens (Morbitzer et al., 2011; Reyon et al., 2012; Weber et al., 2011). In contrast, CRISPR/Cas9 has been widely adopted to endogenously perturb genes (Jinek et al., 2012). However, since enhancers consist of hundreds of base pairs, short indel introduced by one sgRNA alone may not be sufficient to disrupt enhancer activity (He et al., 2011; Reddy et al., 2012). Thus, paired sgRNAs flanking an enhancer have been used for deletion studies. For example, Zhou et al., used this approach to delete putative enhancers of the developmental gene SOX2 in embryonic stem cells (**ESCs**) (Zhou et al., 2014). This study identified multiple enhancers that were essential for regulating SOX2 expression and for the maintenance of pluripotency. To increase the throughput of this approach, Diao et al., generated a pool of sgRNAs spanning 174 potential enhancers to tile the POU5F1 locus in human ESCs (Diao et al., 2017). The authors identified regulators of POU5F1 within hESCs, including enhancers that behaved atypically by only temporarily reducing gene expression when perturbed. By systematically tiling the entire locus, they also found enhancer-like promoters which regulate POU5F1 as well as other genes in the region. Tiling screens can thus uncover a gene’s regulatory elements from a single pooled perturbation experiment. However, CRISPR/Cas9-mediated genetic perturbation of enhancers is limited by two factors: efficiency and scalability. Requiring two sgRNAs to excise an enhancer is inefficient, and often generates a heterogeneous population of cells in which not every cell will harbor an enhancer deletion (Zheng et al., 2014). Clonal selection is also required to ensure homozygous knockout as the cleavage efficiency of both alleles is even lower (Eleveld et al., 2021). Enhancer deletion also complicates high-throughput screens because sgRNA pairs must be simultaneously introduced into an individual cell.

To address these issues, a flexible suite of CRISPR-based tools has been developed to alter enhancer activity through epigenetic modification. This approach fuses catalytically dead Cas9 (**dCas9**) with chromatin modifiers to introduce epigenome edits (Qi et al., 2013). Common dCas9 modifiers include: KRAB, which introduces the heterochromatic modification H3K9me3 to repress enhancer activity (**CRISPRi**); and p300, a histone acetylase that activates enhancer activity (**CRISPRa**) (Gilbert et al., 2013; Hilton et al., 2015). CRISPRi/a has proven to be a potent modifier of enhancer activity and gene expression capable of robust effects even with a single sgRNA (Thakore et al., 2015). This capability has enabled CRISPRi screens with hundreds to thousands of sgRNAs to systematically interrogate multiple enhancers in a single experiment. Fulco et al., applied a tiling CRISPRi screen targeting two loci harboring essential transcription factors (TFs) and enriched them for cells that influence viability (Fulco et al., 2016). This approach identified 9 enhancers which regulate TF expression and harbor phenotypic consequences on cell growth. These experiments indicate that only a subset of enhancers identified by chromatin profiling (Crawford et al., 2006) have impacts on downstream gene expression (Malin et al., 2013). Besides cellular proliferation, RNA expression is another selectable marker that has been applied to CRISPRi enhancer screens (Fulco et al., 2019). CRISPRi-FlowFISH applies an enhancer screen to cells with genes fluorescently labeled using RNA FISH. Sorting cells into bins of expression based on fluorescence intensity identifies enhancers which regulate the labeled genes and the relative level of regulation. Fulco et al., applied CRISPRi-FlowFISH to over 4,000 enhancer-gene pairs to identify the features of enhancer-promoter regulation (Fulco et al., 2019), and showed that enhancers do not always regulate the closest gene. This highlights the importance of chromatin looping and architecture which mediates the DNA-to-DNA interactions mediating enhancer to promoter regulation (Kadauke and Blobel, 2009). One disadvantage of viability and gene expression CRISPRi screens is that the throughput is typically limited to a single phenotypic readout.

### Single-cell screens for enhancer activity in heterogeneous developmental systems

Traditional bulk CRISPR screens are restricted to a single readout that can be assessed. Single-cell RNA-seq (scRNA-seq) has been combined with CRISPR screens to address this limitation by providing high-content transcriptome-wide readouts of cell state (Dixit et al., 2016). In single-cell CRISPR screens, a pool of sgRNAs is transduced into cells followed by scRNA-Seq to detail the transcriptome and perturbations of individual cells. Dixit et al., first demonstrated single-cell CRISPR perturbations (**Perturb-seq**) to knock out dozens of genes and screen for transcriptional differences (Dixit et al., 2016). As Cas9-mediated genetic knockout has low efficiency, Perturb-seq has also been applied in conjunction with dCas9-KRAB in a similar genetic screen (Adamson et al., 2016). Both of these approaches allow for the large-scale screening of multiple elements from a single experiment. This approach has been applied to a developmental system of hESC to cardiomyocyte differentiation, identifying enhancers which impact lineage specification of the cardiac system (Armendariz et al., 2022). Leveraging single-cell gene expression provides not only the transcriptional phenotypes mediated by each enhancer, but also the effect enhancer repression has on cell state. In this way, phenotypes such as differentiation potential can be identified. Similar screens have been applied to promoters in neuronal and endoderm development, yet the application towards enhancers remains limited (Genga et al., 2019; Tian et al., 2019). A significant limitation of using scRNA-seq as a readout is that lowly expressed genes are difficult to detect, which limits statistical analysis and can result in false negatives. To address this problem, TAP-Seq selectively enriches key transcripts in single-cell libraries to increase the sensitivity of detecting genes of interest (Schraivogel et al., 2020). TAP-seq thus increases the detection of genes regulated by targeted enhancers and has the added advantage of reducing sequencing costs. Transcript amplification methods are ideal for enhancer studies as enhancers typically regulate nearby genes, reducing the number of candidate genes for amplification. However, the low overall sensitivity of single-cell RNA-Seq means that very lowly expressed genes may still escape robust detection. Methods to perturb enhancers have rapidly advanced in scale and efficiency over the last decade. Improvements to delivery systems and differentiation models that can better recapitulate development have also provided an increase in disease relevance.

### Functional evaluation of enhancer variants

#### High throughput reporter assays to interpret enhancer variants

While studying enhancers at the element level provides information on their molecular and cellular roles (Figure 1), they lack the resolution of nucleotide variants observed in disease states (Lensch et al., 2022; Thakore et al., 2015). Thus, it is crucial to study perturbations at a nucleotide resolution to elucidate the role of enhancer variants in disease. However, one key challenge is that the effect of a nucleotide variant on an enhancer’s activity is expected to be modest compared to the whole-enhancer perturbations (Kheradpour et al., 2013; Patwardhan et al., 2012). As a result, the molecular changes in target gene expression and downstream cellular phenotypes are also expected to be modest. Thus, sensitive assays are required to measure variant effects (Table 1).

Reporter assays can be readily adapted to study variants by directly incorporating patient-derived nucleotide changes in tested fragments. For example, Smemo et al., identified a TBX5 enhancer G-to-T transversion in a patient with an isolated congenital heart defect. TBX5 is a transcription factor well-studied in the context of cardiac development with coding variants causing the cardiac developmental disorder Holt-Oram syndrome. The wildtype sequence of the enhancer showed myocardium-specific expression in the ventricles and ventricular septum in transgenic mice. Notably, the authors showed abrogation of heart-specific enhancer activity in the G-to-T mutant using beta-galactosidase reporter assay. (Smemo et al., 2012). This study is a rare example validating a patient-derived enhancer variant in a model for developmental disease. However, extending this approach more widely requires higher throughput methods.

To increase the throughput of the variants and elements in the reporter assays, saturation mutagenesis with MPRA has been used to study many disease-associated variants at a single nucleotide resolution (Kircher et al., 2019). In this study, the authors examined over 30,000 single nucleotide variants for 20 disease-associated regulatory elements. The authors identified developmentally relevant enhancer variants, including the well-known zone of polarizing activity regulatory sequence (ZRS). ZRS is a SHH limb enhancer and the identified variants are known to cause severe limb malformations like polydactyly (Riddle et al., 1993; Zeller et al., 2009). Another example is Factor IX (F9) which is associated with an X-linked bleeding disorder called Hemophilia B Leyden. Single nucleotide changes in the promoter region have been implicated in the disease. Kircher et al discovered mutations in the binding sequences for HNF4A and ETS-related transcription factors that reduced promoter activity.

MPRAs have historically lacked the ability to pinpoint the cell type-specific activity of enhancers, which is critical while studying development. Recent work by Zhao et al., addressed this challenge by developing single-cell MPRA screens. Zhao et al applied this approach in the mouse retina to test 113 variants of the Gnb3 promoter, a cis-regulatory element known for its differential expression among the subtypes of retinal cells. Beyond validating the cell type specificity of Gnb3 promoter a variant identified in a previous study, the authors captured the effects of single nucleotide variants across different binding sites in the promoter in different retinal cells (Murphy et al., 2019; Zhao et al., 2023). For example, the authors created single nucleotide variants in the E-box motif, which is critical for the development of multiple retinal cell subtypes. While most of these variants showed effects on gene expression level, only one variant affected cell type specificity. Thus, scMPRA provides a powerful tool to study the effect of single nucleotide variants in a developmental context in complex tissues.

#### CRISPR genome engineering for analysis of enhancer variants

While CRISPRi is an effective tool for enhancer perturbation at ~1 kb resolution, it is too blunt and cannot resolve function at the level of variant-identified disease conditions.(Lensch et al., 2022; Li et al., 2020; Thakore et al., 2015) Thus, to model the effect of nucleotide variants on enhancer function, alternative tools are needed. The short indels generated by CRISPR/Cas9-directed genome editing (typically <30 base pairs) offer higher resolution than CRISPRi/a (Allen et al., 2018; Koike-Yusa et al., 2014; Kosicki et al., 2022; van Overbeek et al., 2016). This method exploits the ability of the cells to be repaired by a non-homologous end joining pathway and generate random insertions and deletions in the guide RNA targeted region. The first study to successfully characterize enhancer activity using CRISPR/Cas9 studied the human erythroid enhancer of BCL11A (Canver et al., 2015). BCL11A is a developmentally crucial transcriptional repressor that facilitates fetal (HbF) to adult hemoglobin switching (Bauer et al., 2012). Bauer et al identified common genetic variants in the erythroid-specific enhancer of BCL11A that was associated with HbF expression (Bauer et al., 2013). By dissecting the enhancer at near-base-pair resolution using CRISPR/Cas9 saturation mutagenesis, the authors pinpointed nucleotides in the enhancer that altered BCL11A expression and subsequently HbF expression (Canver et al., 2015). Specifically, the authors tiled the enhancers identified using DNAse hypersensitivity assays with gRNAs and quantified the effect of perturbation on target gene HbF, through flow cytometry-based sorting. They identified the effect of enhancer perturbation through guide RNA abundance. Other studies using similar single-gene screening strategies have also functionally identified enhancers at scale (Diao et al., 2017; Sanjana et al., 2016).

One drawback of CRISPR/Cas9 approaches is that the double-strand breaks caused can be detrimental to the cells. Recently developed technologies like base editing have solved this problem. For example, Martin-Rufino et al applied base editing screens to precisely alter variants in the promoter region of the γ-hemoglobin gene (HBG1/2), an important component of fetal hemoglobin (Martin-Rufino et al., 2023). Using a pooled base editing screen for ~120 sgRNAs targeting the 300 bp promoter, coupled with FACS-based enrichment for HbF populations, the authors identified new and previously known variants that increase HbF expression. Importantly, the authors also performed pooled single-cell genotyping to link the nucleotide variants to the phenotype. In a separate study, Chen et al., took a tiered approach by combining deep learning-based methodologies with CRISPRi and base editing screens to identify variants in enhancers of CD69 (Chen et al., 2023). Finally, Morris et al., extended this base editing screening strategy to functionally test GWAS variants for blood traits with a single-cell RNA-Seq readout (Morris et al., 2023). Besides base editing, other methods like prime editing, which is currently being used to study variants in protein-coding regions, can be also applied to study enhancer variants (Erwood et al., 2022).

One challenge to studying enhancer variants is the ability to map variants to phenotypes. In the BCL11A enhancer dissection study, the authors sorted the cells based on target gene expression (HbF) and quantified gRNAs contributing to that phenotype. While this enables the study of perturbations at scale, it is an indirect measure for actual edits on DNA. Variant-level analyses that measure nucleotide edits at scale have been recently applied to study exons. Findlay et al., used saturation mutagenesis to study 13 exons of BRCA1 to characterize variants of unknown significance (Findlay et al., 2018). This study used homology-directed repair to supply all possible single nucleotide variants and quantified the effects using growth-based screens. Since the authors sequenced the installed variants in genomic DNA and measured the effects of the variants at the RNA level, this study offers a comprehensive way to link variants to gene expression. Extending this approach to non-coding elements in a disease-relevant system can offer a way to map enhancers’ effect on target genes at variant resolution.

### Future challenges

**Figure 2.**
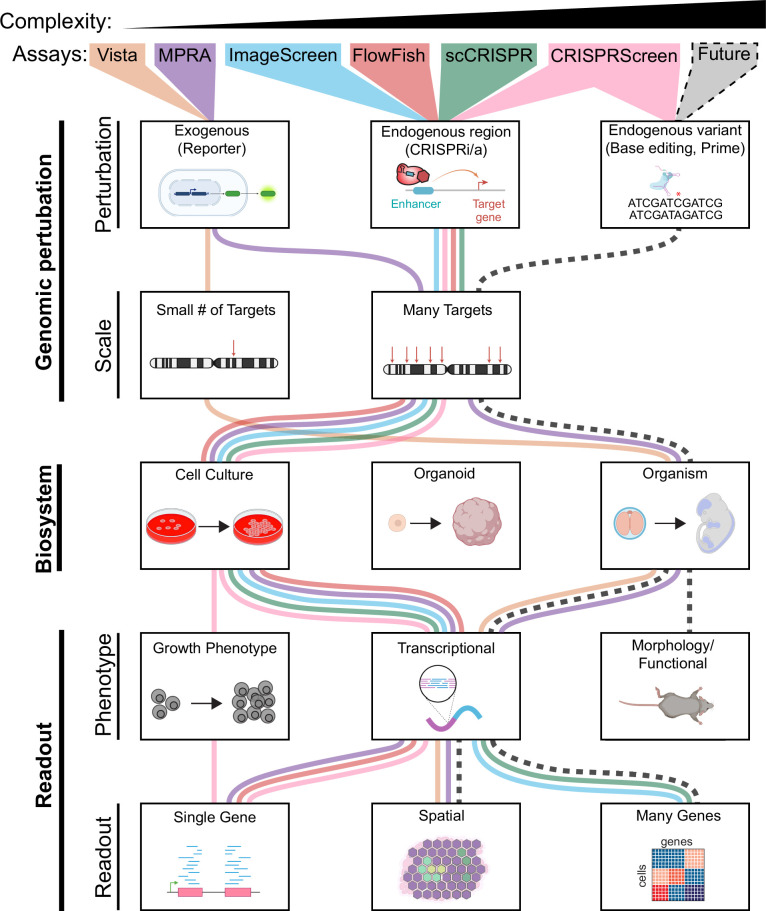
Features of enhancer perturbation studies. Studies of enhancer variants in developmental disease have been carried out using diverse genomic perturbation methods, biosystems, and readouts. Each of these three layers can be organized into increasing levels of complexity with increasing biomedical relevance. We envision that future technologies (black dashed line) will enable analyses of higher complexity.

GWAS indicate that ~90% of disease-associated variants are non-coding and likely at enhancers (Edwards et al., 2013). However, to date, the vast majority of enhancers and their phenotype-associated genetic variants remain uncharacterized. Thus, to understand the molecular and cellular mechanisms of developmental diseases, a key future challenge will be the development of new technologies that enable more comprehensive functional studies of enhancers and their genetic variants. We view the spectrum of these genomic technologies along three dimensions: the resolution of genomic perturbations, the complexity of the cellular system, and the depth of the readout (Figure 2). Here, we evaluate current technologies along each of these dimensions and discuss future challenges.

#### Genomic perturbation: From elements to variants

MPRAs enable the high-throughput study of enhancer function at base-pair resolution but lack endogenous context. Conversely, CRISPRi/a studies enable medium-scale perturbation of enhancers in their native endogenous context, but lack the resolution to give insights on base-level variants sequenced in patients (Armendariz et al., 2022; Fulco et al., 2019; Fulco et al., 2016; Xie et al., 2017). One recent development is to employ base editing or prime editing to test the molecular and cellular phenotypes of endogenously installed variants. These tools are already being used to study disease-relevant protein coding regions (Erwood et al., 2022). A key future goal will be to gain comprehensive analysis of enhancers, with both endogenous context and base-pair resolution. This could be attained by a combination of multiple technologies (CRISPRi/a for endogenous context and MPRA for base-pair resolution), or by scalable base/prime editing approaches. However, one complication could be enhancer redundancy in which multiple enhancers or variants cooperatively regulate a given gene. Notable examples indicate that variants in multiple enhancers may jointly contribute to disease development (Cannavò et al., 2016; Corradin et al., 2014; Corradin and Scacheri, 2014; Kvon et al., 2021; Osterwalder et al., 2018). Combinatorial perturbation of multiple enhancers will require more efficient perturbation systems and would add significant complexity to functional analyses.

#### Cell systems: From cell lines to organoids to animal models

Existing enhancer studies have largely focused on static in vitro systems, especially immortalized or primary cell lines (Fulco et al., 2019). However, the temporal-specific activity of enhancers requires dynamic biological systems to model development and capture the impact of enhancers on developmental phenotypes. One approach taken is to perform CRISPRi studies in human pluripotent stem cells (hPSCs) during differentiation (Armendariz et al., 2022; Genga et al., 2019; Wu et al., 2022). One key challenge is that, since enhancers are time-dependent, understanding how they work together to establish and maintain gene expression networks could require a dense sampling of time points (Bonn et al., 2012).

More complex biological systems like human organoids and mouse models, which better recapitulate development, are needed to study the spatiotemporal impacts of enhancers. Organoids are currently available for a variety of developmental systems including the brain, kidney, and heart (Di Lullo and Kriegstein, 2017; Lewis-Israeli et al., 2021; Nishinakamura, 2019). One advantage is that these systems represent 3D models of human development. A CRISPRi screen has been applied to an organoid model, identifying key TFs in fetal lung development through perturbation of select promoters (Sun et al., 2022). Similar 3D systems could be used to study the role of enhancers in developmental disease. In vivo systems are currently able to interrogate individual enhancers and variants (Smemo et al., 2012). While in vivo studies offer the highest level of disease relevance, they suffer from low throughput. As a result, they are best employed to characterize enhancers which have been prioritized using screens in simpler models. While in vivo CRISPR screens have been accomplished, perturbation complexity remains a key challenge (Gemberling et al., 2021; Jin et al., 2020). A delivery system is needed that can endogenously perturb enhancers with high efficiency that can capture enhancer activity at the appropriate developmental time point. However, the number of cells available for analysis at relevant developmental stages may be limiting. Until this critical issue is addressed, organoid models will be a more tractable system for perturbation screens in the near future. A tiered approach with multiple systems of increasing complexity could also serve as a bridge until newer technologies are developed.

#### Readouts: From expression to morphology to function

Interpreting the function of an enhancer or its variants is limited by the sensitivity and scalability of the phenotype measured. There are many phenotypes that can be measured in cells, including growth, transcription, and morphology. Measuring gene expression is a common readout for many enhancer studies (Armendariz et al., 2022; Canver et al., 2015; Diao et al., 2017; Fulco et al., 2016; Reilly et al., 2021). Gene expression readouts offer a trade-off between the scalability and sensitivity of the genes measured. RNA sequencing-based approaches can provide an unbiased measurement of the transcriptome. However, detecting lowly expressed genes remains a challenge, especially at the single-cell level. This is especially relevant for transcription factors, which are often lowly or moderately expressed (Pokhilko et al., 2021). On the other hand, direct RNA labeling through fluorescent in-situ hybridization (**FISH**) is more sensitive. For example, FISH-based methods have been adapted to identify regulatory elements of key genes by perturbing enhancers and sorting cells based on fluorescence intensity after RNA labeling (Fulco et al., 2019; Reilly et al., 2021). One drawback is that FISH-based approaches can only survey a handful of pre-identified genes. More recent approaches including MerFISH, seqFISH, osmFISH, and others can increase the scale of genes detected, but are significantly more challenging to establish (Codeluppi et al., 2018; De Biase et al., 2021; Eng et al., 2019; Xia et al., 2019). Similarly, targeted transcript amplification has been developed to enrich genes of interest (Schraivogel et al., 2020). These approaches and improved single-cell chemistry will likely increase the sensitivity of transcript detection.

While gene expression readouts are common, more complex phenotypes could offer different insights into disease states. Morphological and functional readouts are two such phenotypes. Morphology as a phenotype has been enabled by advances in imaging. For example, Cell Painting is a multiplexed image-based assay for the detection of cellular organelles like mitochondria, Golgi apparatus, and cytoskeleton through fluorescent dyes (Bray et al., 2016). Subtle perturbation phenotypes such as size, shape, and structure can then be interrogated across multiple cellular components. In combination with CRISPR perturbations, optical screens can provide information on organelle localization, cell morphology, and cell-cell interactions (Feldman et al., 2019). Optical CRISPR screens have been employed to characterize multiple phenotypic features of perturbation of essential genes (Funk et al., 2022). Since optical screens are compatible with live-cell imaging, combining them with enhancer perturbation can provide insights into the cellular dynamics across lineage specification during development. Beyond morphology, enhancers and their variants can lead to other organismal and physiological phenotypes. For example, Cunningham et al., identified two enhancers of Tbx5 that are not essential for limb development through genetic knockout (Cunningham et al., 2018). However, such phenotypes are not amenable to high throughput characterization (Bender et al., 2000; Cunningham et al., 2018; Johnson et al., 2012). Several functional assays have been developed which incorporate high-throughput techniques for large-scale screening. Patch-seq is one such readout that combines whole-cell electrophysiological recordings with single-cell RNA sequencing and immunochemistry (Cadwell et al., 2016; Fuzik et al., 2016). Such multimodal readouts will enable the linking of perturbations to gene expression and functional phenotype. Such analyses will yield fuller insights into an enhancer’s role in disease and development.

#### Predictive modeling

Since there are millions of regulatory elements in the human genome and many more sequence variants, it is not feasible to experimentally test all of them for their impact on developmental disorders. Computational models that can accurately predict the regulatory activity of sequence variants will be an essential component to close this gap. Several recent advances highlight the promise of this nascent field. For example, Sei is a sequence-based deep learning model trained on a compendium of ~20,000 epigenetic features, including open chromatin and transcription factor binding from ~1300 cell lines and tissues (Chen et al., 2022). Given a DNA sequence, Sei accurately predicts whether the sequence is a regulatory element and in what cell contexts. Since Sei models sequences, it can also predict how genetic variants alter enhancer activity. Similarly, Enformer also applies deep learning to predict gene regulatory activity from genomic sequences (Avsec et al., 2021). One key advance of Enformer is the ability to use information from distal genomic interactions (~100 kb) to improve predictions. In this way, Enformer can predict long-range interactions between promoters and enhancers.

Predictive modeling is an exciting area for future research, and there are many areas for future improvement. First, current approaches have focused on predicting molecular phenotypes including expression and epigenetic status. Future needs include the accurate modeling of how biological networks and pathways are perturbed by sequence variants, as well as more complex cellular and organismal phenotypes. Second, accurate predictions rely on good training data. However, much of our existing training data is derived from cell lines. Epigenetic data from human developmental systems, especially in vivo, are still rare. In addition, since there is limited data on systematic perturbation studies, existing models will need to improve as these data become available. Active learning strategies with deep integration of experimental and modeling components will be crucial to guide experiments to where computational modeling can be most improved. Third, predictive models need to be accurate across diverse populations, and doing so requires the ability to model the effect of genetic background. Fourth, the development of explainable AI models will offer insights into these models and the features important for prediction (Novakovsky et al., 2023). We anticipate that improved predictive modeling capabilities will drive clinical applications to interpret the molecular, cellular, and organismal impact of newly identified variants in patients.

### Conclusion

From the initial discovery of enhancers in SV40 four decades ago, the field has witnessed a rapid progression of tools to characterize enhancers and their variants in development (Banerji et al., 1981). The 2000s saw the development of advanced reporter systems to characterize enhancer activity in vivo and culminated in the development of MPRAs (Patwardhan et al., 2012; Patwardhan et al., 2009). The 2010s witnessed the comprehensive mapping of enhancers, as well as new genome engineering tools to perturb enhancers endogenously at scale, both at increasingly cellular resolution. In the coming decade, we anticipate that innovative technologies will spearhead the high-throughput characterization of how developmental enhancers and their genetic variants impact molecular and cellular phenotypes in vivo (Figure 2). However, to gain comprehensive views of all enhancers at the nucleotide and cellular resolution, experimental strategies alone will not be sufficient. Predictive modeling and machine learning approaches will be instrumental to achieve this goal. Ultimately, this knowledge will enable the interpretation of enhancer variants in both research and clinical settings.

## References

[bib1] Adamson B, Norman TM, Jost M, Cho MY, Nuñez JK, Chen Y, Villalta JE, Gilbert LA, Horlbeck MA, Hein MY, Pak RA, Gray AN, Gross CA, Dixit A, Parnas O, Regev A, Weissman JS (2016). A Multiplexed Single-Cell CRISPR Screening Platform Enables Systematic Dissection of the Unfolded Protein Response. Cell.

[bib2] Allen F, Crepaldi L, Alsinet C, Strong AJ, Kleshchevnikov V, De Angeli P, Páleníková P, Khodak A, Kiselev V, Kosicki M, Bassett AR, Harding H, Galanty Y, Muñoz-Martínez F, Metzakopian E, Jackson SP, Parts L (2018). Predicting the mutations generated by repair of Cas9-induced double-strand breaks. Nature Biotechnology.

[bib3] Armendariz DA, Goetsch SC, Sundarrajan A, Sivakumar S, Wang Y, Xie S, Munshi N, Hon GC (2022). CHD-Associated Enhancers Shape Human Cardiomyocyte Lineage Commitment. bioRxiv.

[bib4] Arnold CD, Gerlach D, Stelzer C, Boryń ŁM, Rath M, Stark A (2013). Genome-wide quantitative enhancer activity maps identified by STARR-seq. Science.

[bib5] Avsec Ž, Agarwal V, Visentin D, Ledsam JR, Grabska-Barwinska A, Taylor KR, Assael Y, Jumper J, Kohli P, Kelley DR (2021). Effective gene expression prediction from sequence by integrating long-range interactions. Nature Methods.

[bib6] Banerji J, Rusconi S, Schaffner W (1981). Expression of a beta-globin gene is enhanced by remote SV40 DNA sequences. Cell.

[bib7] Basak A, Hancarova M, Ulirsch JC, Balci TB, Trkova M, Pelisek M, Vlckova M, Muzikova K, Cermak J, Trka J, Dyment DA, Orkin SH, Daly MJ, Sedlacek Z, Sankaran VG (2015). BCL11A deletions result in fetal hemoglobin persistence and neurodevelopmental alterations. The Journal of Clinical Investigation.

[bib8] Bauer DE, Kamran SC, Orkin SH (2012). Reawakening fetal hemoglobin: prospects for new therapies for the β-globin disorders. Blood.

[bib9] Bauer DE, Kamran SC, Lessard S, Xu J, Fujiwara Y, Lin C, Shao Z, Canver MC, Smith EC, Pinello L, Sabo PJ, Vierstra J, Voit RA, Yuan GC, Porteus MH, Stamatoyannopoulos JA, Lettre G, Orkin SH (2013). An erythroid enhancer of BCL11A subject to genetic variation determines fetal hemoglobin level. Science.

[bib10] Bender MA, Bulger M, Close J, Groudine M (2000). Beta-globin gene switching and DNase I sensitivity of the endogenous beta-globin locus in mice do not require the locus control region. Molecular Cell.

[bib11] Boch J, Scholze H, Schornack S, Landgraf A, Hahn S, Kay S, Lahaye T, Nickstadt A, Bonas U (2009). Breaking the code of DNA binding specificity of TAL-type III effectors. Science.

[bib12] Bonn S, Zinzen RP, Girardot C, Gustafson EH, Perez-Gonzalez A, Delhomme N, Ghavi-Helm Y, Wilczyński B, Riddell A, Furlong EEM (2012). Tissue-specific analysis of chromatin state identifies temporal signatures of enhancer activity during embryonic development. Nature Genetics.

[bib13] Bray MA, Singh S, Han H, Davis CT, Borgeson B, Hartland C, Kost-Alimova M, Gustafsdottir SM, Gibson CC, Carpenter AE (2016). Cell Painting, a high-content image-based assay for morphological profiling using multiplexed fluorescent dyes. Nature Protocols.

[bib14] Bruneau BG, Nemer G, Schmitt JP, Charron F, Robitaille L, Caron S, Conner DA, Gessler M, Nemer M, Seidman CE, Seidman JG (2001). A murine model of Holt-Oram syndrome defines roles of the T-box transcription factor Tbx5 in cardiogenesis and disease. Cell.

[bib15] Cadwell CR, Palasantza A, Jiang X, Berens P, Deng Q, Yilmaz M, Reimer J, Shen S, Bethge M, Tolias KF, Sandberg R, Tolias AS (2016). Electrophysiological, transcriptomic and morphologic profiling of single neurons using Patch-seq. Nature Biotechnology.

[bib16] Cannavò E, Khoueiry P, Garfield DA, Geeleher P, Zichner T, Gustafson EH, Ciglar L, Korbel JO, Furlong EEM (2016). Shadow enhancers are pervasive features of developmental regulatory networks. Current Biology.

[bib17] Canver MC, Smith EC, Sher F, Pinello L, Sanjana NE, Shalem O, Chen DD, Schupp PG, Vinjamur DS, Garcia SP, Luc S, Kurita R, Nakamura Y, Fujiwara Y, Maeda T, Yuan GC, Zhang F, Orkin SH, Bauer DE (2015). BCL11A enhancer dissection by Cas9-mediated in situ saturating mutagenesis. Nature.

[bib18] Chen KM, Wong AK, Troyanskaya OG, Zhou J (2022). A sequence-based global map of regulatory activity for deciphering human genetics. Nature Genetics.

[bib19] Chen Z, Javed N, Moore M, Wu J, Sun G, Vinyard M, Collins A, Pinello L, Najm FJ, Bernstein BE (2023). Integrative dissection of gene regulatory elements at base resolution. Cell Genomics.

[bib20] Civelek M, Wu Y, Pan C, Raulerson CK, Ko A, He A, Tilford C, Saleem NK, Stančáková A, Scott LJ, Fuchsberger C, Stringham HM, Jackson AU, Narisu N, Chines PS, Small KS, Kuusisto J, Parks BW, Pajukanta P, Kirchgessner T, Collins FS, Gargalovic PS, Boehnke M, Laakso M, Mohlke KL, Lusis AJ (2017). Genetic regulation of adipose gene expression and cardio-metabolic traits. American Journal of Human Genetics.

[bib21] Codeluppi S, Borm LE, Zeisel A, La Manno G, van Lunteren JA, Svensson CI, Linnarsson S (2018). Spatial organization of the somatosensory cortex revealed by osmFISH. Nature Methods.

[bib22] Conrad SE, Botchan MR (1982). Isolation and characterization of human DNA fragments with nucleotide sequence homologies with the simian virus 40 regulatory region. Molecular and Cellular Biology.

[bib23] Corradin O, Saiakhova A, Akhtar-Zaidi B, Myeroff L, Willis J, Cowper-Sal lari R, Lupien M, Markowitz S, Scacheri PC (2014). Combinatorial effects of multiple enhancer variants in linkage disequilibrium dictate levels of gene expression to confer susceptibility to common traits. Genome Research.

[bib24] Corradin O, Scacheri PC (2014). Enhancer variants: evaluating functions in common disease. Genome Medicine.

[bib25] Crawford GE, Holt IE, Whittle J, Webb BD, Tai D, Davis S, Margulies EH, Chen Y, Bernat JA, Ginsburg D, Zhou D, Luo S, Vasicek TJ, Daly MJ, Wolfsberg TG, Collins FS (2006). Genome-wide mapping of DNase hypersensitive sites using massively parallel signature sequencing (MPSS). Genome Research.

[bib26] Cunningham TJ, Lancman JJ, Berenguer M, Dong PDS, Duester G (2018). Genomic knockout of two presumed forelimb tbx5 enhancers reveals they are nonessential for limb development. Cell Reports.

[bib27] Cusanovich DA, Hill AJ, Aghamirzaie D, Daza RM, Pliner HA, Berletch JB, Filippova GN, Huang X, Christiansen L, DeWitt WS, Lee C, Regalado SG, Read DF, Steemers FJ, Disteche CM, Trapnell C, Shendure J (2018a). A single-cell atlas of in vivo mammalian chromatin accessibility. Cell.

[bib28] Cusanovich DA, Reddington JP, Garfield DA, Daza RM, Aghamirzaie D, Marco-Ferreres R, Pliner HA, Christiansen L, Qiu X, Steemers FJ, Trapnell C, Shendure J, Furlong EEM (2018b). The cis-regulatory dynamics of embryonic development at single-cell resolution. Nature.

[bib29] De Biase D, Prisco F, Piegari G, Ilsami A, d’Aquino I, Baldassarre V, Zito Marino F, Franco R, Papparella S, Paciello O (2021). RNAScope *in situ* Hybridization as a Novel Technique for the Assessment of c-KIT mRNA Expression in Canine Mast Cell Tumor. Frontiers in Veterinary Science.

[bib30] Diao Y, Fang R, Li B, Meng Z, Yu J, Qiu Y, Lin KC, Huang H, Liu T, Marina RJ, Jung I, Shen Y, Guan KL, Ren B (2017). A tiling-deletion-based genetic screen for cis-regulatory element identification in mammalian cells. Nature Methods.

[bib31] Di Lullo E, Kriegstein AR (2017). The use of brain organoids to investigate neural development and disease. Nature Reviews. Neuroscience.

[bib32] Dixit A, Parnas O, Li B, Chen J, Fulco CP, Jerby-Arnon L, Marjanovic ND, Dionne D, Burks T, Raychowdhury R, Adamson B, Norman TM, Lander ES, Weissman JS, Friedman N, Regev A (2016). Perturb-Seq: Dissecting Molecular Circuits with Scalable Single-Cell RNA Profiling of Pooled Genetic Screens. Cell.

[bib33] Domcke S, Hill AJ, Daza RM, Cao J, O’Day DR, Pliner HA, Aldinger KA, Pokholok D, Zhang F, Milbank JH, Zager MA, Glass IA, Steemers FJ, Doherty D, Trapnell C, Cusanovich DA, Shendure J (2020). A human cell atlas of fetal chromatin accessibility. Science.

[bib34] Edwards SL, Beesley J, French JD, Dunning AM (2013). Beyond GWASs: illuminating the dark road from association to function. American Journal of Human Genetics.

[bib35] Eleveld TF, Bakali C, Eijk PP, Stathi P, Vriend LE, Poddighe PJ, Ylstra B (2021). Engineering large-scale chromosomal deletions by CRISPR-Cas9. Nucleic Acids Research.

[bib36] Eng CHL, Lawson M, Zhu Q, Dries R, Koulena N, Takei Y, Yun J, Cronin C, Karp C, Yuan GC, Cai L (2019). Transcriptome-scale super-resolved imaging in tissues by RNA seqFISH. Nature.

[bib37] Erwood S, Bily TMI, Lequyer J, Yan J, Gulati N, Brewer RA, Zhou L, Pelletier L, Ivakine EA, Cohn RD (2022). Saturation variant interpretation using CRISPR prime editing. Nature Biotechnology.

[bib38] Fahed AC, Gelb BD, Seidman JG, Seidman CE (2013). Genetics of congenital heart disease: the glass half empty. Circulation Research.

[bib39] Feldman D, Singh A, Schmid-Burgk JL, Carlson RJ, Mezger A, Garrity AJ, Zhang F, Blainey PC (2019). Optical pooled screens in human cells. Cell.

[bib40] Findlay GM, Daza RM, Martin B, Zhang MD, Leith AP, Gasperini M, Janizek JD, Huang X, Starita LM, Shendure J (2018). Accurate classification of BRCA1 variants with saturation genome editing. Nature.

[bib41] Fulco CP, Munschauer M, Anyoha R, Munson G, Grossman SR, Perez EM, Kane M, Cleary B, Lander ES, Engreitz JM (2016). Systematic mapping of functional enhancer-promoter connections with CRISPR interference. Science.

[bib42] Fulco CP, Nasser J, Jones TR, Munson G, Bergman DT, Subramanian V, Grossman SR, Anyoha R, Doughty BR, Patwardhan TA, Nguyen TH, Kane M, Perez EM, Durand NC, Lareau CA, Stamenova EK, Aiden EL, Lander ES, Engreitz JM (2019). Activity-by-contact model of enhancer-promoter regulation from thousands of CRISPR perturbations. Nature Genetics.

[bib43] Funk L, Su KC, Ly J, Feldman D, Singh A, Moodie B, Blainey PC, Cheeseman IM (2022). The phenotypic landscape of essential human genes. Cell.

[bib44] Fuzik J, Zeisel A, Máté Z, Calvigioni D, Yanagawa Y, Szabó G, Linnarsson S, Harkany T (2016). Integration of electrophysiological recordings with single-cell RNA-seq data identifies neuronal subtypes. Nature Biotechnology.

[bib45] Gao T, Zheng Z, Pan Y, Zhu C, Wei F, Yuan J, Sun R, Fang S, Wang N, Zhou Y, Qian J (2022). scEnhancer: a single-cell enhancer resource with annotation across hundreds of tissue/cell types in three species. Nucleic Acids Research.

[bib46] Gasperini M, Tome JM, Shendure J (2020). Towards a comprehensive catalogue of validated and target-linked human enhancers. Nature Reviews. Genetics.

[bib47] Gemberling MP, Siklenka K, Rodriguez E, Tonn-Eisinger KR, Barrera A, Liu F, Kantor A, Li L, Cigliola V, Hazlett MF, Williams CA, Bartelt LC, Madigan VJ, Bodle JC, Daniels H, Rouse DC, Hilton IB, Asokan A, Ciofani M, Poss KD, Reddy TE, West AE, Gersbach CA (2021). Transgenic mice for in vivo epigenome editing with CRISPR-based systems. Nature Methods.

[bib48] Genga RMJ, Kernfeld EM, Parsi KM, Parsons TJ, Ziller MJ, Maehr R (2019). Single-Cell RNA-Sequencing-Based CRISPRi Screening Resolves Molecular Drivers of Early Human Endoderm Development. Cell Reports.

[bib49] Gilbert LA, Larson MH, Morsut L, Liu Z, Brar GA, Torres SE, Stern-Ginossar N, Brandman O, Whitehead EH, Doudna JA, Lim WA, Weissman JS, Qi LS (2013). CRISPR-mediated modular RNA-guided regulation of transcription in eukaryotes. Cell.

[bib50] Gusev A, Lee SH, Neale BM, Trynka G, Vilhjalmsson BJ, Finucane H, Xu H, Zang C, Ripke S, Stahl E, Schizophrenia Working Group of the PGC n, SWE-SCZ Consortium n, Kahler AK, Hultman CM, Purcell SM, McCarroll SA, Daly M, Pasaniuc B, Sullivan PF, Wray NR, Raychaudhuri S, Price AL (2014). Regulatory Variants Explain Much More Heritability than Coding Variants across 11 Common Diseases. bioRxiv.

[bib51] Hammer RE, Swift GH, Ornitz DM, Quaife CJ, Palmiter RD, Brinster RL, MacDonald RJ (1987). The rat elastase I regulatory element is an enhancer that directs correct cell specificity and developmental onset of expression in transgenic mice. Molecular and Cellular Biology.

[bib52] He A, Kong SW, Ma Q, Pu WT (2011). Co-occupancy by multiple cardiac transcription factors identifies transcriptional enhancers active in heart. PNAS.

[bib53] Hilton IB, D’Ippolito AM, Vockley CM, Thakore PI, Crawford GE, Reddy TE, Gersbach CA (2015). Epigenome editing by a CRISPR-Cas9-based acetyltransferase activates genes from promoters and enhancers. Nature Biotechnology.

[bib54] Inoue F, Ahituv N (2015). Decoding enhancers using massively parallel reporter assays. Genomics.

[bib55] Inoue F, Kreimer A, Ashuach T, Ahituv N, Yosef N (2019). Identification and massively parallel characterization of regulatory elements driving neural induction. Cell Stem Cell.

[bib56] Jin X, Simmons SK, Guo A, Shetty AS, Ko M, Nguyen L, Jokhi V, Robinson E, Oyler P, Curry N, Deangeli G, Lodato S, Levin JZ, Regev A, Zhang F, Arlotta P (2020). In vivo Perturb-Seq reveals neuronal and glial abnormalities associated with autism risk genes. Science.

[bib57] Jinek M, Chylinski K, Fonfara I, Hauer M, Doudna JA, Charpentier E (2012). A programmable dual-RNA-guided DNA endonuclease in adaptive bacterial immunity. Science.

[bib58] Johnson JE, Wold BJ, Hauschka SD (1989). Muscle creatine kinase sequence elements regulating skeletal and cardiac muscle expression in transgenic mice. Molecular and Cellular Biology.

[bib59] Johnson KD, Hsu AP, Ryu MJ, Wang J, Gao X, Boyer ME, Liu Y, Lee Y, Calvo KR, Keles S, Zhang J, Holland SM, Bresnick EH (2012). Cis-element mutated in GATA2-dependent immunodeficiency governs hematopoiesis and vascular integrity. The Journal of Clinical Investigation.

[bib60] Kadauke S, Blobel GA (2009). Chromatin loops in gene regulation. Biochimica et Biophysica Acta.

[bib61] Kheradpour P, Ernst J, Melnikov A, Rogov P, Wang L, Zhang X, Alston J, Mikkelsen TS, Kellis M (2013). Systematic dissection of regulatory motifs in 2000 predicted human enhancers using a massively parallel reporter assay. Genome Research.

[bib62] Kircher M, Xiong C, Martin B, Schubach M, Inoue F, Bell RJA, Costello JF, Shendure J, Ahituv N (2019). Saturation mutagenesis of twenty disease-associated regulatory elements at single base-pair resolution. Nature Communications.

[bib63] Klein JC, Agarwal V, Inoue F, Keith A, Martin B, Kircher M, Ahituv N, Shendure J (2020). A systematic evaluation of the design and context dependencies of massively parallel reporter assays. Nature Methods.

[bib64] Kleinjan DA, van Heyningen V (2005). Long-range control of gene expression: emerging mechanisms and disruption in disease. American Journal of Human Genetics.

[bib65] Koike-Yusa H, Li Y, Tan EP, Velasco-Herrera MDC, Yusa K (2014). Genome-wide recessive genetic screening in mammalian cells with a lentiviral CRISPR-guide RNA library. Nature Biotechnology.

[bib66] Korkmaz G, Lopes R, Ugalde A (2016). Functional genetic screens for enhancer elements in the human genome using CRISPR-Cas9. Nat Biotechnol.

[bib67] Kosicki M, Allen F, Steward F, Tomberg K, Pan Y, Bradley A (2022). Cas9-induced large deletions and small indels are controlled in a convergent fashion. Nature Communications.

[bib68] Kvon EZ, Zhu Y, Kelman G, Novak CS, Plajzer-Frick I, Kato M, Garvin TH, Pham Q, Harrington AN, Hunter RD, Godoy J, Meky EM, Akiyama JA, Afzal V, Tran S, Escande F, Gilbert-Dussardier B, Jean-Marçais N, Hudaiberdiev S, Ovcharenko I, Dobbs MB, Gurnett CA, Manouvrier-Hanu S, Petit F, Visel A, Dickel DE, Pennacchio LA (2020). Comprehensive in vivo interrogation reveals phenotypic impact of human enhancer variants. Cell.

[bib69] Kvon EZ, Waymack R, Gad M, Wunderlich Z (2021). Enhancer redundancy in development and disease. Nature Reviews. Genetics.

[bib70] Lalanne JB, Regalado SG, Domcke S, Calderon D, Martin B, Li T, Suiter CC, Lee C, Trapnell C, Shendure J (2022). Multiplex Profiling of Developmental Enhancers with Quantitative, Single-Cell Expression Reporters. bioRxiv.

[bib71] Lambert JT, Su-Feher L, Cichewicz K, Warren TL, Zdilar I, Wang Y, Lim KJ, Haigh JL, Morse SJ, Canales CP, Stradleigh TW, Castillo Palacios E, Haghani V, Moss SD, Parolini H, Quintero D, Shrestha D, Vogt D, Byrne LC, Nord AS (2021). Parallel functional testing identifies enhancers active in early postnatal mouse brain. eLife.

[bib72] Lensch S, Herschl MH, Ludwig CH, Sinha J, Hinks MM, Mukund A, Fujimori T, Bintu L (2022). Dynamic spreading of chromatin-mediated gene silencing and reactivation between neighboring genes in single cells. eLife.

[bib73] Lettice LA, Heaney SJH, Purdie LA, Li L, de Beer P, Oostra BA, Goode D, Elgar G, Hill RE, de Graaff E (2003). A long-range Shh enhancer regulates expression in the developing limb and fin and is associated with preaxial polydactyly. Human Molecular Genetics.

[bib74] Lettice LA, Devenney P, De Angelis C, Hill RE (2017). The conserved sonic hedgehog limb enhancer consists of discrete functional elements that regulate precise spatial expression. Cell Reports.

[bib75] Lewis-Israeli YR, Wasserman AH, Gabalski MA, Volmert BD, Ming Y, Ball KA, Yang W, Zou J, Ni G, Pajares N, Chatzistavrou X, Li W, Zhou C, Aguirre A (2021). Self-assembling human heart organoids for the modeling of cardiac development and congenital heart disease. Nature Communications.

[bib76] Li R, Xia X, Wang X, Sun X, Dai Z, Huo D, Zheng H, Xiong H, He A, Wu X (2020). Generation and validation of versatile inducible CRISPRi embryonic stem cell and mouse model. PLOS Biology.

[bib77] Lu L, Liu X, Huang W-K, Giusti-Rodríguez P, Cui J, Zhang S, Xu W, Wen Z, Ma S, Rosen JD, Xu Z, Bartels CF, Kawaguchi R, Hu M, Scacheri PC, Rong Z, Li Y, Sullivan PF, Song H, Ming G-L, Li Y, Jin F (2020). Robust hi-c maps of enhancer-promoter interactions reveal the function of non-coding genome in neural development and diseases. Molecular Cell.

[bib78] Lungu-Mitea S, Lundqvist J (2020). Potentials and pitfalls of transient in vitro reporter bioassays: interference by vector geometry and cytotoxicity in recombinant zebrafish cell lines. Archives of Toxicology.

[bib79] Malin J, Aniba MR, Hannenhalli S (2013). Enhancer networks revealed by correlated DNAse hypersensitivity states of enhancers. Nucleic Acids Research.

[bib80] Martin-Rufino JD, Castano N, Pang M, Grody EI, Joubran S, Caulier A, Wahlster L, Li T, Qiu X, Riera-Escandell AM, Newby GA, Al’Khafaji A, Chaudhary S, Black S, Weng C, Munson G, Liu DR, Wlodarski MW, Sims K, Oakley JH, Fasano RM, Xavier RJ, Lander ES, Klein DE, Sankaran VG (2023). Massively parallel base editing to map variant effects in human hematopoiesis. Cell.

[bib81] McDermott DA, Bressan MC, He J, Lee JS, Aftimos S, Brueckner M, Gilbert F, Graham GE, Hannibal MC, Innis JW, Pierpont ME, Raas-Rothschild A, Shanske AL, Smith WE, Spencer RH, St John-Sutton MG, van Maldergem L, Waggoner DJ, Weber M, Basson CT (2005). TBX5 genetic testing validates strict clinical criteria for Holt-Oram syndrome. Pediatric Research.

[bib82] Melnikov A, Murugan A, Zhang X, Tesileanu T, Wang L, Rogov P, Feizi S, Gnirke A, Callan CG, Kinney JB, Kellis M, Lander ES, Mikkelsen TS (2012). Systematic dissection and optimization of inducible enhancers in human cells using a massively parallel reporter assay. Nature Biotechnology.

[bib83] Morbitzer R, Elsaesser J, Hausner J, Lahaye T (2011). Assembly of custom TALE-type DNA binding domains by modular cloning. Nucleic Acids Research.

[bib84] Morris JA, Caragine C, Daniloski Z, Domingo J, Barry T, Lu L, Davis K, Ziosi M, Glinos DA, Hao S, Mimitou EP, Smibert P, Roeder K, Katsevich E, Lappalainen T, Sanjana NE (2023). Discovery of target genes and pathways at GWAS loci by pooled single-cell CRISPR screens. Science.

[bib85] Moscou MJ, Bogdanove AJ (2009). A simple cipher governs DNA recognition by TAL effectors. Science.

[bib86] Murphy DP, Hughes AE, Lawrence KA, Myers CA, Corbo JC (2019). *Cis*-regulatory basis of sister cell type divergence in the vertebrate retina. eLife.

[bib87] Nishinakamura R (2019). Human kidney organoids: progress and remaining challenges. Nature Reviews. Nephrology.

[bib88] Novakovsky G, Dexter N, Libbrecht MW, Wasserman WW, Mostafavi S (2023). Obtaining genetics insights from deep learning via explainable artificial intelligence. Nature Reviews Genetics.

[bib89] Osterwalder M, Barozzi I, Tissières V, Fukuda-Yuzawa Y, Mannion BJ, Afzal SY, Lee EA, Zhu Y, Plajzer-Frick I, Pickle CS, Kato M, Garvin TH, Pham QT, Harrington AN, Akiyama JA, Afzal V, Lopez-Rios J, Dickel DE, Visel A, Pennacchio LA (2018). Enhancer redundancy provides phenotypic robustness in mammalian development. Nature.

[bib90] Parker SCJ, Stitzel ML, Taylor DL, Orozco JM, Erdos MR, Akiyama JA, van Bueren KL, Chines PS, Narisu N, Black BL, Visel A, Pennacchio LA, Collins FS, NISC Comparative Sequencing Program, National Institutes of Health Intramural Sequencing Center Comparative Sequencing Program Authors, NISC Comparative Sequencing Program Authors (2013). Chromatin stretch enhancer states drive cell-specific gene regulation and harbor human disease risk variants. PNAS.

[bib91] Patwardhan RP, Lee C, Litvin O, Young DL, Pe’er D, Shendure J (2009). High-resolution analysis of DNA regulatory elements by synthetic saturation mutagenesis. Nature Biotechnology.

[bib92] Patwardhan RP, Hiatt JB, Witten DM, Kim MJ, Smith RP, May D, Lee C, Andrie JM, Lee SI, Cooper GM, Ahituv N, Pennacchio LA, Shendure J (2012). Massively parallel functional dissection of mammalian enhancers in vivo. Nature Biotechnology.

[bib93] Pennacchio LA, Ahituv N, Moses AM, Prabhakar S, Nobrega MA, Shoukry M, Minovitsky S, Dubchak I, Holt A, Lewis KD, Plajzer-Frick I, Akiyama J, De Val S, Afzal V, Black BL, Couronne O, Eisen MB, Visel A, Rubin EM (2006). In vivo enhancer analysis of human conserved non-coding sequences. Nature.

[bib94] Pinkert CA, Ornitz DM, Brinster RL, Palmiter RD (1987). An albumin enhancer located 10 kb upstream functions along with its promoter to direct efficient, liver-specific expression in transgenic mice. Genes & Development.

[bib95] Pokhilko A, Handel AE, Curion F, Volpato V, Whiteley ES, Bøstrand S, Newey SE, Akerman CJ, Webber C, Clark MB, Bowden R, Cader MZ (2021). Targeted single-cell RNA sequencing of transcription factors enhances the identification of cell types and trajectories. Genome Research.

[bib96] Qi LS, Larson MH, Gilbert LA, Doudna JA, Weissman JS, Arkin AP, Lim WA (2013). Repurposing CRISPR as an RNA-guided platform for sequence-specific control of gene expression. Cell.

[bib97] Razin SV, Ulianov SV, Iarovaia OV (2023). Enhancer Function in the 3D Genome. Genes.

[bib98] Reddy TE, Gertz J, Pauli F, Kucera KS, Varley KE, Newberry KM, Marinov GK, Mortazavi A, Williams BA, Song L, Crawford GE, Wold B, Willard HF, Myers RM (2012). Effects of sequence variation on differential allelic transcription factor occupancy and gene expression. Genome Research.

[bib99] Reilly SK, Gosai SJ, Gutierrez A, Mackay-Smith A, Ulirsch JC, Kanai M, Mouri K, Berenzy D, Kales S, Butler GM, Gladden-Young A, Bhuiyan RM, Stitzel ML, Finucane HK, Sabeti PC, Tewhey R (2021). Author Correction: Direct characterization of cis-regulatory elements and functional dissection of complex genetic associations using HCR-FlowFISH. Nature Genetics.

[bib100] Reyon D, Tsai SQ, Khayter C, Foden JA, Sander JD, Joung JK (2012). FLASH assembly of TALENs for high-throughput genome editing. Nature Biotechnology.

[bib101] Riddle RD, Johnson RL, Laufer E, Tabin C (1993). Sonic hedgehog mediates the polarizing activity of the ZPA. Cell.

[bib102] Romanov SE, Kalashnikova DA, Laktionov PP (2021). Methods of massive parallel reporter assays for investigation of enhancers. Vavilov Journal of Genetics and Breeding.

[bib103] Ron G, Globerson Y, Moran D, Kaplan T (2017). Promoter-enhancer interactions identified from Hi-C data using probabilistic models and hierarchical topological domains. Nature Communications.

[bib104] Sanjana NE, Wright J, Zheng K, Shalem O, Fontanillas P, Joung J, Cheng C, Regev A, Zhang F (2016). High-resolution interrogation of functional elements in the noncoding genome. Science.

[bib105] Schraivogel D, Gschwind AR, Milbank JH, Leonce DR, Jakob P, Mathur L, Korbel JO, Merten CA, Velten L, Steinmetz LM (2020). Targeted Perturb-seq enables genome-scale genetic screens in single cells. Nature Methods.

[bib106] Shlyueva D, Stampfel G, Stark A (2014). Transcriptional enhancers: from properties to genome-wide predictions. Nature Reviews. Genetics.

[bib107] Smemo S, Campos LC, Moskowitz IP, Krieger JE, Pereira AC, Nobrega MA (2012). Regulatory variation in a TBX5 enhancer leads to isolated congenital heart disease. Human Molecular Genetics.

[bib108] Smemo S, Tena JJ, Kim KH, Gamazon ER, Sakabe NJ, Gómez-Marín C, Aneas I, Credidio FL, Sobreira DR, Wasserman NF, Lee JH, Puviindran V, Tam D, Shen M, Son JE, Vakili NA, Sung HK, Naranjo S, Acemel RD, Manzanares M, Nagy A, Cox NJ, Hui CC, Gomez-Skarmeta JL, Nóbrega MA (2014). Obesity-associated variants within FTO form long-range functional connections with IRX3. Nature.

[bib109] Smith RP, Taher L, Patwardhan RP, Kim MJ, Inoue F, Shendure J, Ovcharenko I, Ahituv N (2013). Massively parallel decoding of mammalian regulatory sequences supports a flexible organizational model. Nature Genetics.

[bib110] Stanojevic D, Small S, Levine M (1991). Regulation of a segmentation stripe by overlapping activators and repressors in the *Drosophila* embryo. Science.

[bib111] Sun D, Batlle OL, van den Ameele J, Thomas JC, He P, Lim K, Tang W, Xu C, Meyer KB, Teichmann SA, Marioni JC, Jackson SP, Brand AH, Rawlins EL (2022). An Organoid CRISPRi Screen Revealed That SOX9 Primes Human Fetal Lung Tip Progenitors to Receive WNT and RTK Signals. bioRxiv.

[bib112] Swift GH, Hammer RE, MacDonald RJ, Brinster RL (1984). Tissue-specific expression of the rat pancreatic elastase I gene in transgenic mice. Cell.

[bib113] Thakore PI, D’Ippolito AM, Song L, Safi A, Shivakumar NK, Kabadi AM, Reddy TE, Crawford GE, Gersbach CA (2015). Highly specific epigenome editing by CRISPR-Cas9 repressors for silencing of distal regulatory elements. Nature Methods.

[bib114] Tian R, Gachechiladze MA, Ludwig CH, Laurie MT, Hong JY, Nathaniel D, Prabhu AV, Fernandopulle MS, Patel R, Abshari M, Ward ME, Kampmann M (2019). CRISPR Interference-Based Platform for Multimodal Genetic Screens in Human iPSC-Derived Neurons. Neuron.

[bib115] van Overbeek M, Capurso D, Carter MM, Thompson MS, Frias E, Russ C, Reece-Hoyes JS, Nye C, Gradia S, Vidal B, Zheng J, Hoffman GR, Fuller CK, May AP (2016). DNA Repair Profiling Reveals Nonrandom Outcomes at Cas9-Mediated Breaks. Molecular Cell.

[bib116] Vermunt MW, Zhang D, Blobel GA (2019). The interdependence of gene-regulatory elements and the 3D genome. The Journal of Cell Biology.

[bib117] Visel A, Minovitsky S, Dubchak I, Pennacchio LA (2007). VISTA Enhancer Browser--a database of tissue-specific human enhancers. Nucleic Acids Research.

[bib118] Wang S, Wen F, Tessneer KL, Gaffney PM (2016). TALEN-mediated enhancer knockout influences TNFAIP3 gene expression and mimics a molecular phenotype associated with systemic lupus erythematosus. Genes and Immunity.

[bib119] Weber E, Gruetzner R, Werner S, Engler C, Marillonnet S, Bendahmane M (2011). Assembly of designer TAL effectors by Golden Gate cloning. PLOS ONE.

[bib120] Williamson I, Lettice LA, Hill RE, Bickmore WA (2016). Shh and ZRS enhancer colocalisation is specific to the zone of polarising activity. Development.

[bib121] Wu D, Poddar A, Ninou E, Hwang E, Cole MA, Liu SJ, Horlbeck MA, Chen J, Replogle JM, Carosso GA, Eng NWL, Chang J, Shen Y, Weissman JS, Lim DA (2022). Dual genome-wide coding and lncRNA screens in neural induction of induced pluripotent stem cells. Cell Genomics.

[bib122] Xia C, Fan J, Emanuel G, Hao J, Zhuang X (2019). Spatial transcriptome profiling by MERFISH reveals subcellular RNA compartmentalization and cell cycle-dependent gene expression. PNAS.

[bib123] Xie S, Duan J, Li B, Zhou P, Hon GC (2017). Multiplexed engineering and analysis of combinatorial enhancer activity in single cells. Molecular Cell.

[bib124] Zeller R, López-Ríos J, Zuniga A (2009). Vertebrate limb bud development: moving towards integrative analysis of organogenesis. Nature Reviews. Genetics.

[bib125] Zhao S, Hong CKY, Myers CA, Granas DM, White MA, Corbo JC, Cohen BA (2023). A single-cell massively parallel reporter assay detects cell-type-specific gene regulation. Nature Genetics.

[bib126] Zheng Q, Cai X, Tan MH, Schaffert S, Arnold CP, Gong X, Chen CZ, Huang S (2014). Precise gene deletion and replacement using the CRISPR/Cas9 system in human cells. BioTechniques.

[bib127] Zhou HY, Katsman Y, Dhaliwal NK, Davidson S, Macpherson NN, Sakthidevi M, Collura F, Mitchell JA (2014). A Sox2 distal enhancer cluster regulates embryonic stem cell differentiation potential. Genes & Development.

